# Case report: Sequential complement inhibition and BAFF/APRIL blockade in progressive IgA nephropathy and IgA vasculitis nephritis: a report of two cases

**DOI:** 10.3389/fimmu.2026.1866835

**Published:** 2026-07-08

**Authors:** Xinyuan Tian, Wanyin Hou, Xiaojuan Yu, Lei Jiang, Xin Zhang, Xu Zhang, Suxia Wang, Ying Tan, Lijun Liu

**Affiliations:** 1 Department of Nephrology, Peking University First Hospital, Peking University Institute of Nephrology, Beijing, China; 2 Key Laboratory of Renal Disease, Ministry of Health, Beijing, China; 3 Key Laboratory of Chronic Kidney Disease Prevention and Treatment (Peking University), Ministry of Education, Beijing, China; 4 Research Units of Diagnosis and Treatment of Immune-mediated Kidney Diseases, Chinese Academy of Medical Sciences, Beijing, China; 5 Electron Microscopy Laboratory, Peking University First Hospital, Beijing, China

**Keywords:** complement inhibition, eculizumab, IgA nephropathy, IgA vasculitis nephritis, iptacopan, telitacicept

## Abstract

Rapidly progressive IgA nephropathy (IgAN) and IgA vasculitis nephritis (IgAVN) are characterized by marked glomerular inflammation, extensive extracapillary proliferation, and rapid renal function decline. Despite increasing insights into their shared pathogenic mechanisms, optimal treatment strategies remain uncertain. Here, we report two patients with rapidly progressive crescentic disease, one with primary IgAN and one with IgAVN, who received sequential complement inhibition combined with BAFF/APRIL blockade. Both patients showed substantial reductions in proteinuria and stabilization of kidney function during follow-up. These cases suggest that multi-pathway targeted therapy may represent a promising approach for selected patients with aggressive IgAN or IgAVN.

## Background

Rapidly progressive (crescentic) IgA nephropathy (IgAN) is a high-risk clinical phenotype associated with rapid deterioration of kidney function and poor renal outcomes. IgA vasculitis nephritis (IgAVN), which shares substantial pathogenic overlap with IgAN, may exhibit similar aggressive clinical and histopathologic features (1–3). In both conditions, renal injury is thought to result from the deposition of galactose-deficient IgA1 (Gd-IgA1)-containing immune complexes and activation of the complement system (4, 5). In recent years, therapies targeting key pathogenic pathways, including complement activation and BAFF/APRIL-mediated B-cell responses, have shown encouraging clinical results in IgAN. These advances support the exploration of multi-pathway targeted therapy.

## Case presentation

### Case 1

A 74-year-old woman had a 19-year history of microscopic hematuria and proteinuria. During follow-up, the urine protein-to-creatinine ratio ranged from 0.6 to 1.0 g/gCr, with normal renal function (Scr 59–74 μmol/L). Twenty days before admission, she developed severe bilateral lower-extremity edema and was hospitalized locally; Scr increased from 166 to 189 μmol/L, with a rapid decline in eGFR. Her history included hypertension (treated with sacubitril/valsartan and benidipine) and depression (treated with escitalopram). Ten days before admission, gastroscopy showed chronic atrophic gastritis with erosions and a duodenal bulb ulcer. There was no family history of kidney disease.

On admission, blood pressure was 159/88 mmHg, with marked bilateral lower-extremity edema. Laboratory tests showed nephrotic-range proteinuria (5.72 g/day), microscopic hematuria (10–20 RBCs/HPF), Scr 187 μmol/L, and hypoalbuminemia (24.9 g/L). Additional findings included serum uric acid 457 μmol/L, iPTH 67.01 pg/mL, and mildly elevated IgA (4.02 g/L). Complement C3/C4 levels were normal, and HbA1c was 5.4%. No monoclonal protein was detected on serum or urine immunofixation. Autoimmune serologies (ANA, anti-dsDNA, anti-ENA, ANCA, anti-GBM, anti-PLA2R antibodies) were negative, and hepatitis B showed no active infection. Renal ultrasound revealed bilaterally small kidneys with poor corticomedullary differentiation.

**Figure 1:**
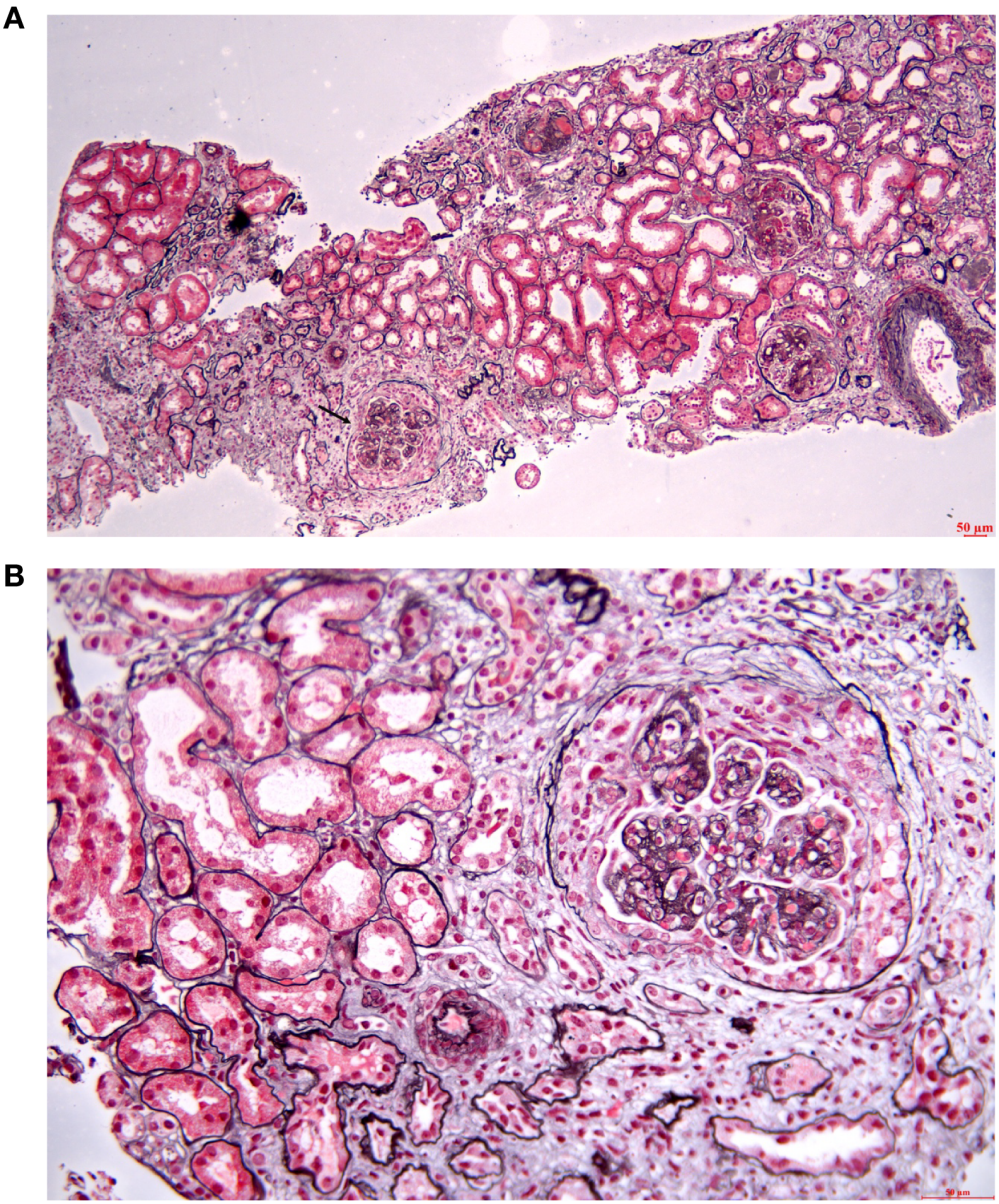
Renal biopsy findings in case 1 (PASM staining). **(A)** Low-power view of a cellular (arrow) crescent and moderate mesangial proliferation (×50). **(B)** High-power view of the cellular crescent in Figure 1A (×200). .

Renal biopsy was performed. Immunofluorescence showed strong granular mesangial staining for IgA (4+) and C3 (3+), weak IgG (+), and negative IgM and C1q. Kappa and lambda light chains showed equal mesangial staining (3+). Fibrinogen and C4d were negative. Light microscopy identified 33 glomeruli, of which 4 were globally sclerotic and 13 were ischemic (51%). The remaining glomeruli exhibited mesangial and endocapillary hypercellularity, diffuse matrix expansion and mesangial fuchsinophilic deposits. Capillary walls were thickened with segmental double-contour formation. Two cellular crescents (6%) were present, along with one segmental glomerulosclerosis lesion and four adhesions. The tubulointerstitium showed tubular injury, multifocal atrophy, interstitial inflammation, and fibrosis. Arterioles demonstrated wall thickening with fibrous intimal hyperplasia (**Figure 1**). Electron microscopy revealed mesangial electron-dense deposits and diffuse podocyte foot process effacement. Overall, findings support IgA nephropathy with a membranoproliferative pattern (Oxford classification: M1E1S1T1C1).

**Figure 2:**
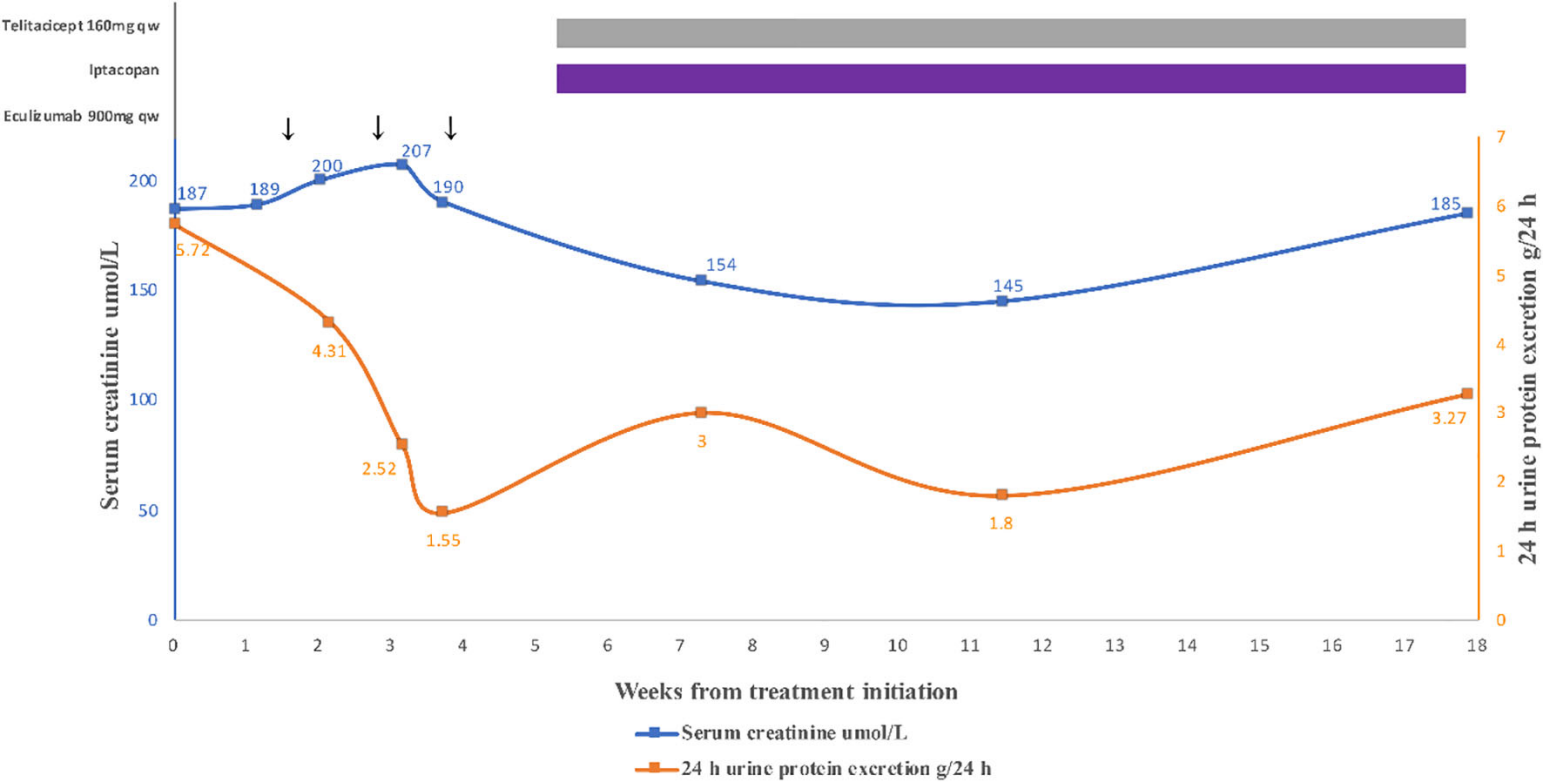
Time course of renal parameters and treatment interventions in case 1. Serum creatinine levels (blue line, left y-axis) and 24-hour urinary protein excretion (orange line, right y-axis) are shown over time, expressed as weeks from initiation of therapy. The upper panel illustrates the timing and duration of therapeutic interventions, including telitacicept, iptacopan, and eculizumab. Arrows indicate individual doses of eculizumab (900 mg weekly).

In view of rapidly progressive renal dysfunction, nephrotic-range proteinuria, and crescent formation, immunosuppressive therapy was warranted. However, due to active peptic ulcer disease, glucocorticoids were avoided. Considering prominent C3 deposition, complement-targeted therapy was initiated with eculizumab (900 mg weekly for three doses), followed by iptacopan (200 mg twice daily) and telitacicept (160 mg weekly) (**Figure 2**). Supportive care included diuretics and albumin, angiotensin receptor blockade, management of anemia and hyperuricemia, and intensive blood pressure and lipid control. Prophylactic antibiotics and vaccination against Neisseria meningitidis and Streptococcus pneumoniae were administered.

After three doses of eculizumab, proteinuria decreased from 5.72 g to 2.52 g and then 1.55 g. Serum creatinine stabilized with modest improvement. With subsequent iptacopan and telitacicept therapy, Scr further decreased to 145–154 μmol/L. Over ~4 months of follow-up, proteinuria remained reduced (1.8–3.27 g/day), and serum albumin increased to 32 g/L. No infectious complications were observed.

### Case 2

A 37-year-old man was initially diagnosed with Henoch–Schönlein purpura (HSP) 24 years ago, presenting with purpura, abdominal pain, and proteinuria, which achieved complete remission after corticosteroid therapy. Nine years ago, mild proteinuria was detected and treated with losartan. Seven years ago, proteinuria increased to 2.5 g/day with normal renal function (Scr 73 μmol/L); renal biopsy at that time showed HSP nephropathy (class IIIb), and prednisone was initiated but discontinued after 2 months. One year prior to admission, serum creatinine rose to 150 μmol/L. One week before admission, he developed left-leg edema with further deterioration of renal function (Scr 387 μmol/L) and elevated iPTH (151 pg/mL). His medical history was notable for prior glioma resection with residual hemiplegia and epilepsy, and there was no family history of kidney disease.

On admission, blood pressure was 157/93 mmHg with only mild lower limb edema and no active rash or purpura. Urinalysis showed nephrotic-range proteinuria (6.89 g/day) and microscopic hematuria (25–30 RBCs/HPF). Serum IgA and complement levels were normal. Serologic tests for ANCA, anti-GBM, ANA, and anti-dsDNA were negative, with no monoclonal protein detected. Renal ultrasound showed normal-sized kidneys with increased cortical echogenicity.

**Figure 3:**
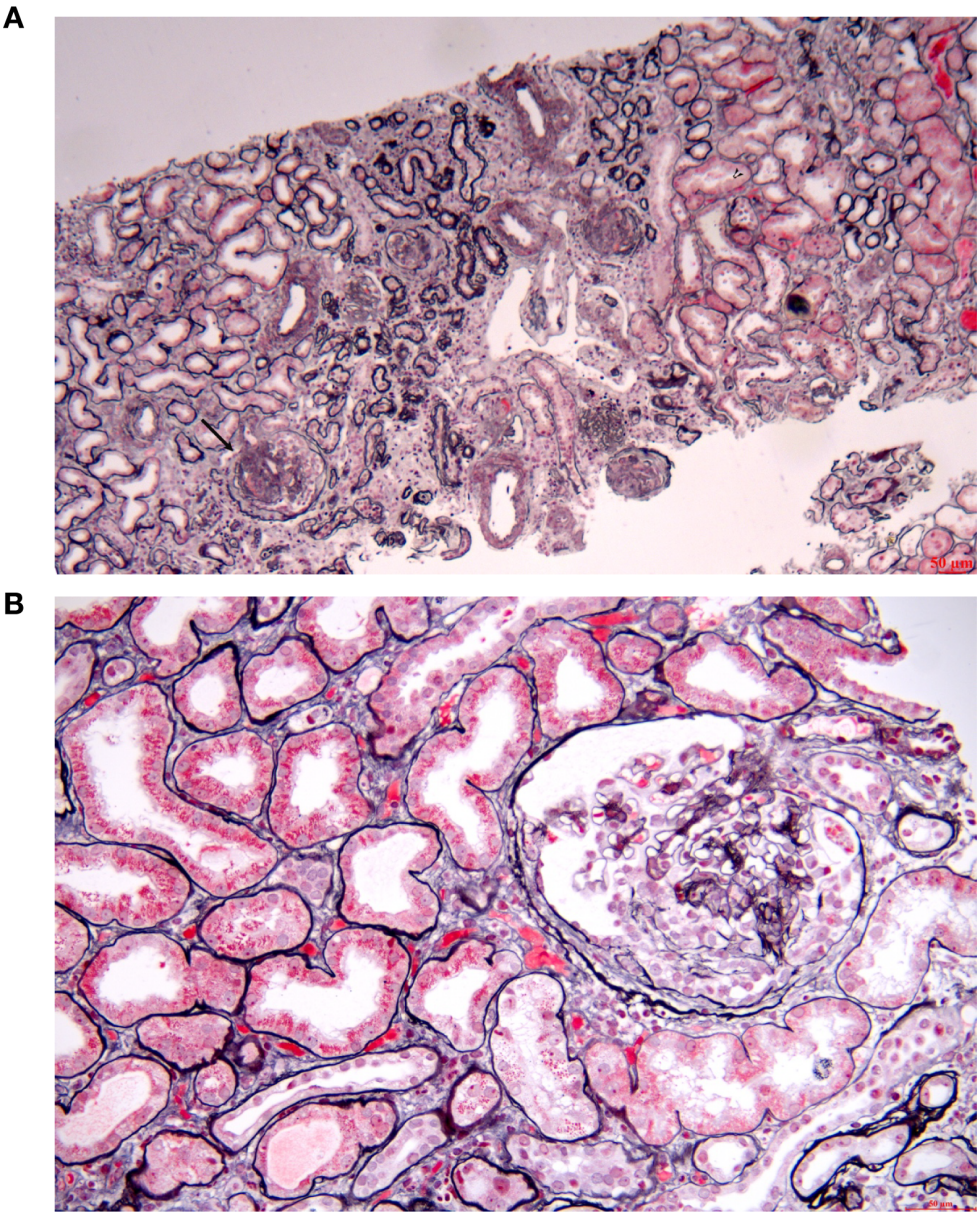
Renal biopsy findings in case 2 (PASM staining). **(A)** Low-power view showing a fibrocellular crescent (arrow) crescent (×50). **(B)** High-power view of a cellular crescent (×200).

Renal biopsy was performed on the day of admission. Immunofluorescence microscopy demonstrated dominant mesangial staining for IgA (3+) and C3 (4+), with no staining for IgG, IgM, and C1q. Both kappa and lambda light chains showed comparable mesangial staining (2+). Fibrin-related antigen staining was weakly positive. Immunohistochemistry revealed weak C4d positivity. Light microscopy showed significant glomerular injury. Of 19 glomeruli, 8 (42%) were globally sclerotic and 2 (11%) had ischemic sclerosis. The remaining glomeruli exhibited mild segmental mesangial hypercellularity, matrix expansion, increased endocapillary cellularity, and mesangial fuchsinophilic deposits. Crescents were present in 6 glomeruli (32%), including 1 cellular and 5 fibrocellular crescents, along with segmental glomerulosclerosis. The tubulointerstitium demonstrated tubular epithelial injury, multifocal atrophy, interstitial inflammation, and mild fibrosis. Arterioles showed wall thickening with hyaline change and focal intimal edema (**Figure 3**). Electron microscopy showed electron-dense deposits in the mesangial areas, along with diffuse podocyte foot process effacement. Histopathologic findings were consistent with IgA nephropathy, with both active and chronic lesions (Oxford classification: M0E1S1T1C2). Based on the patient’s well-documented history of HSP, the current episode was considered progressive IgA vasculitis nephritis.

**Figure 4:**
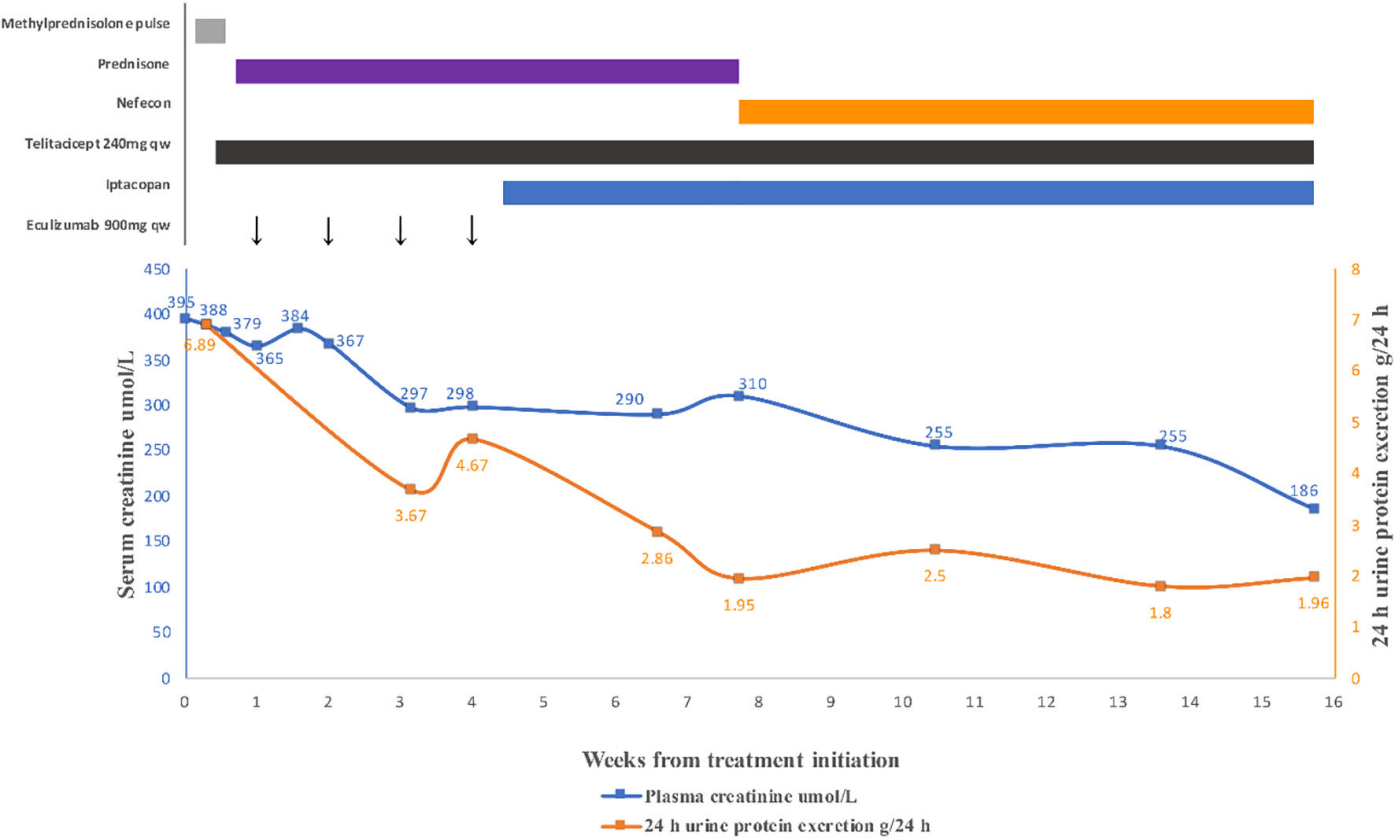
Time course of renal parameters and treatment interventions in case 2. Serum creatinine levels (blue line, left y-axis) and 24-hour urinary protein excretion (orange line, right y-axis) are shown over time, expressed as weeks from initiation of therapy. The upper panel illustrates the timing and duration of therapeutic interventions, including methylprednisolone pulse therapy, oral prednisone, targeted-release budesonide (Nefecon), telitacicept, iptacopan, and eculizumab. Arrows indicate individual doses of eculizumab (900 mg weekly).

Given the rapid rise in serum creatinine and crescentic lesions on biopsy, high-dose methylprednisolone pulse therapy (0.5 g/day for 3 days) was initiated, followed by oral prednisone (30 mg/day). Eculizumab (900 mg weekly) and telitacicept (240 mg weekly) were started early, leading to initial stabilization of renal function, with Scr decreasing from 395 to 367 μmol/L within two weeks. After three doses, renal parameters improved markedly: Scr declined to 297 μmol/L, 24-hour proteinuria decreased from 6.89 to 3.67 g (**Figure 4**). Prophylactic oral amoxicillin was administered during eculizumab treatment. After four doses of eculizumab and vaccination against Neisseria meningitidis and Streptococcus pneumoniae, iptacopan (200 mg twice daily) was initiated as sequential therapy. During maintenance therapy, systemic prednisone was gradually tapered, and targeted-release budesonide (Nefecon) was introduced as a steroid-sparing strategy. Renal function continued to improve during this period. At ~16 weeks, Scr decreased to 186 μmol/L and proteinuria remained around 2.0 g/day. No infectious complications were observed.

## Discussion

IgAN is the most common primary glomerulonephritis worldwide and a major cause of kidney failure in young adults (6). Its pathogenesis is multifactorial, involving production of pathogenic IgA1, immune complex formation, mesangial activation, and complement activation within renal tissue (7). IgAVN shares key pathogenic features with IgAN, including involvement of Gd-IgA1, immune complex deposition, and complement activation, suggesting overlapping disease mechanisms that contribute to progressive renal injury (1–3). Despite advances in supportive and immunosuppressive therapies, a subset of patients still develops rapidly progressive disease characterized by persistent inflammation and sustained complement activation (7). These mechanistic insights have driven the development of targeted therapies, including BAFF/APRIL inhibition with telitacicept to reduce pathogenic IgA production and immune complex formation, and complement inhibitors such as iptacopan and eculizumab to attenuate downstream inflammatory injury, supporting a therapeutic strategy that simultaneously targets IgA dysregulation and complement activation in progressive IgAN (8–10).

In this case report, both patients had biopsy-proven IgA-dominant crescentic lesions with prominent mesangial C3 deposition. Although one patient had primary IgAN and the other had IgAVN, the shared finding of extensive mesangial C3 deposition suggested complement activation and provided a rationale for complement-targeted therapy. Accordingly, eculizumab was initiated early and was associated with stabilization of kidney function and a marked reduction in proteinuria. Treatment was subsequently transitioned to iptacopan and combined with telitacicept as a sequential strategy targeting both complement activation and upstream IgA dysregulation. Renal function and proteinuria continued to improve during follow-up, without serious infectious complications.

Of note, in the second case, high-dose methylprednisolone followed by oral prednisone was administered at treatment initiation because of active crescentic lesions, the patient’s relatively young age, and the absence of contraindications to corticosteroid therapy. This treatment likely contributed to the early clinical improvement; therefore, the initial stabilization of kidney function cannot be attributed solely to complement inhibition. Targeted-release budesonide (Nefecon) was introduced during glucocorticoid tapering after the patient had shown a favorable response to initial corticosteroid therapy, resulting in overlap with the telitacicept treatment phase. Because both therapies may influence pathogenic IgA production, their individual contributions cannot be clearly distinguished. Nevertheless, renal function continued to improve following the introduction of Nefecon.

A major limitation of this study is the inability to determine the independent contribution of each therapeutic intervention. Given the sequential administration of multiple therapies, including systemic glucocorticoids, complement inhibition, telitacicept, and budesonide, the specific effects of each individual treatment cannot be clearly separated.

Our cases highlight the potential value of a multi-pathway therapeutic strategy targeting both complement activation and upstream IgA immune dysregulation in patients with aggressive IgAN or IgAVN. Future prospective studies, including combination and head-to-head trials, are warranted to define optimal patient selection, treatment sequencing, and long-term outcomes.

## Conclusion

These cases suggest that a multi-target therapeutic approach addressing both complement activation and pathogenic IgA production may improve outcomes in progressive IgA nephropathy or IgA vasculitis nephritis.

## Patient perspective

During follow-up, both patients reported satisfaction with the stabilization or improvement of their kidney disease and expressed willingness to continue long-term monitoring and treatment.

## Data Availability

The original contributions presented in the study are included in the article/supplementary material. Further inquiries can be directed to the corresponding author.
